# CTLs heterogeneity and plasticity: implications for cancer immunotherapy

**DOI:** 10.1186/s12943-024-01972-6

**Published:** 2024-03-21

**Authors:** Shengkun Peng, Anqi Lin, Aimin Jiang, Cangang Zhang, Jian Zhang, Quan Cheng, Peng Luo, Yifeng Bai

**Affiliations:** 1 Department of Radiology, Sichuan Provincial People’s Hospital, School of Medicine, University of Electronic Science and Technology of China, Chengdu, China; 2 Department of Oncology, Zhujiang Hospital, Southern Medical University, Guangzhou, 510282 Guangdong China; 3 Department of Urology, Changhai hospital, Naval Medical University (Second Military Medical University), Shanghai, China; 4 Department of Pathogenic Microbiology and ImmunologySchool of Basic Medical Sciences, Xi’an Jiaotong University, Xi’an, 710061 Shaanxi China; 5 Department of Neurosurgery, Xiangya Hospital, Central South University, Changsha, 410008 Hunan China; 6 National Clinical Research Center for Geriatric Disorders, Xiangya HospitalCentral South University, Hunan, China; 7 Department of Oncology, Sichuan Provincial People’s Hospital, School of Medicine, University of Electronic Science and Technology of China, Chengdu, China

**Keywords:** CTLs, Cytotoxic, Tumor immune microenvironment, Biomarkers, Cytotoxic T lymphocytes

## Abstract

Cytotoxic T lymphocytes (CTLs) play critical antitumor roles, encompassing diverse subsets including CD4+, NK, and γδ T cells beyond conventional CD8+ CTLs. However, definitive CTLs biomarkers remain elusive, as cytotoxicity-molecule expression does not necessarily confer cytotoxic capacity. CTLs differentiation involves transcriptional regulation by factors such as T-bet and Blimp-1, although epigenetic regulation of CTLs is less clear. CTLs promote tumor killing through cytotoxic granules and death receptor pathways, but may also stimulate tumorigenesis in some contexts. Given that CTLs cytotoxicity varies across tumors, enhancing this function is critical. This review summarizes current knowledge on CTLs subsets, biomarkers, differentiation mechanisms, cancer-related functions, and strategies for improving cytotoxicity. Key outstanding questions include refining the CTLs definition, characterizing subtype diversity, elucidating differentiation and senescence pathways, delineating CTL-microbe relationships, and enabling multi-omics profiling. A more comprehensive understanding of CTLs biology will facilitate optimization of their immunotherapy applications. Overall, this review synthesizes the heterogeneity, regulation, functional roles, and enhancement strategies of CTLs in antitumor immunity, highlighting gaps in our knowledge of subtype diversity, definitive biomarkers, epigenetic control, microbial interactions, and multi-omics characterization. Addressing these questions will refine our understanding of CTLs immunology to better leverage cytotoxic functions against cancer.

## Introduction

Cytotoxic T lymphocytes (CTLs), as a special type of lymphocyte, play a crucial role in mediating the immune responses responsible for tumor killing and pathogen clearance [1–3]. Due to further exploration of CTLs, the current knowledge of CTLs is not only limited to classical CTLs (e.g., CD8+ CTLs), and CTLs are now also thought to include CD4+ CTLs, γδ-CTLs, and invariant natural killer T (iNK)-CTLs [3]. Studies have shown that different types of CTLs utilize different mechanisms to maximize the recognition and elimination of target cells for the killing of pathogens and tumor cells [3]. CTLs share a wide range of molecular signatures that allow these cells to directly mediate the killing of tumor cells after the recognition of target cells, such as the granule cytosolic pathway (e.g., perforin/granzyme) and the death receptor pathway [e.g., FAS/Fas and Fas ligand (FasL), TNF-related apoptosis-inducing ligand (TRAIL)/TRAIL receptor (TRAIL-R)] [2, 4]. In addition, CTLs may play a further role in tumor elimination by activating other immune cells in the immune system [2].

In recent years, researchers have attempted to identify a set of cellular markers that can directly distinguish CTLs from other immune cells, but a full consensus on these cellular markers has not been reached [5]. In addition to the lack of a fully harmonized set of biomarkers, the following challenges exist with respect to cellular markers for CTLs. For example, lymphocytes that express molecules associated with cytotoxicity do not necessarily exhibit cytotoxicity [5]. Moreover, Jonsson et al. found that even though they express cytotoxicity-associated molecules, GzmK+ GzmB+ CD8 T cells (CD8T_te_K) have a low cytotoxicity potential and are unable to exert cytotoxicity, mainly due to their low levels of granzyme B (GzmB) and perforin, and these cells are thus unable to generate sufficient pores in the plasma membrane of target cells to mediate target cell death [5, 6]. Therefore, cellular markers for CTLs still need to be further explored and discussed in future studies.

Whether CTLs utilize other strategies to kill tumor cells remains unclear, and whether these killing functions and the molecules involved in their processes can be judged as cellular markers for CTLs has not yet been elaborated. Recent studies found that the perforin/granzyme system and the death receptor/ligand system can induce not only apoptosis in target cells but also other types of regulated cell death (RCD), such as necroptosis and pyroptosis [3]. Furthermore, in addition to promoting apoptosis through granzyme-activated caspase activation in the perforin/granzyme system, the subsequent perforin-mediated Ca2+-dependent elevation of reactive oxygen species (ROS) and DNA-damaging processes may play an important role in promoting cell death [1].

The following questions remain poorly addressed in the field of immunotherapy: 1) Can a class of T cells be defined as *cytotoxic* T cells? 2) What are the biomarkers of CTLs? 3) What are the key regulatory molecules involved in the differentiation and development of CTLs? 4) What is the function of CTLs in the tumor immune response? 5) How can the cytotoxic function of CTLs in the tumor immune response be further improved or enhanced? Therefore, in this review, we systematically summarize the major cell types, biomarkers, cell differentiation and development pathways, and biological functions of CTLs. We also systematically describe the currently available information on the targeting of specific pathways to improve or enhance the cytotoxic function of CTLs.

## Classifications of CTLs

In recent decades, the knowledge of CTLs has been limited because these are a class of T lymphocytes with cytotoxic functions against tumor cells, and CTLs are effector T cells that develop from activated naïve CD8+ T cells to exert tumor-killing functions [7]. The structure of the polarization between CTLs and their target cells serves as the basis through which CTLs exert their cytotoxic function, which ultimately leads to the death of the target cells [7]. In recent years, with the development of high-throughput sequencing, especially single-cell transcriptome sequencing, mass spectrometry flow, and single-cell level sequencing analysis of T-cell receptor (TCR), the understanding of CTLs has not only been limited to CD8+ T cells with cytotoxic function but also CD4+ T cells with cytotoxicity, NKT cells and γδ T cells.

### CD8+ CTLs

CD8+ CTLs are cytotoxic effector cells that differentiate after the initial activation of CD8+ T cells, are essential for tumor cell clearance, and play an important role in the killing of pathogens (such as viruses and bacteria) [8, 9]. Upon exposure to antigens presented by antigen-presenting cells (APCs), antigen-specific naïve CD8+ T cells are activated and enter a process of clonal proliferation to become CD8+ CTLs with the ability to secrete inflammatory cytokines and cytotoxic molecules [10, 11]. One of the main characteristics of CD8+ CTLs is that they are highly reactive to target cells, including virally infected cells and tumor cells. One of the key features of CD8+ CTLs is their potent killing ability against target cells, including virally infected cells and tumor cells. CD8+ CTLs can directly use a suite of effector molecules, including granzymes, perforin, and the FAS/FASL pathway, to execute their killing effects on target cells. The importance of CD8+ CTLs as a type of CTL has been described in detail in previous studies [12].

### CD4+ CTLs

Traditionally, CD4+ T cells play important functions mainly in antibody production, helper antigen-specific CD8+ T-cell activation, and immunomodulation. Cytotoxic CD4+ T cells were first identified in allogeneic immune rejection, but this phenomenon has now long been regarded as an artifact produced by in vitro culture [13]. In recent years, several studies have revealed that CD4+ CTLs are widely present in humans and mice [14]. CD4+ CTLs can kill target cells through the major histocompatibility complex (MHC)-II-like molecule-dependent recognition of target cells, the secretion of cytotoxic substances (e.g., granzymes and perforins), or the death-ligand receptor pathway [14]. Subsequently CD4+ CTLs were further shown to play important roles in viral infections [14], tumors [14–16], autoimmune diseases [14, 17], and vaccinations [5, 18], among other processes. CD4+ CTLs represent an independent subset of CD4+ T helper (Th) cells with antigen-specific cytotoxic functions [14]. Currently, all known CD4+ Th subpopulations, including regulatory T cells (Tregs), type 1 regulatory cells (Tr1s), Th1, Th2, Th17, and nonclassical subpopulations, exhibit cytotoxic potential [5, 19].

### γδ-CTLs

Γδ T cells are mainly found in barrier tissues such as skin and mucous membranes and account for only a small fraction of CD3+ T cells in peripheral circulation and tissues. γδ T cells enter their activation state within minutes after antigenic stimulation [20]. Due to their ability to rapidly produce a variety of cytokines after activation, γδ T cells are involved in creating the first line of defense against infections and tumors [21, 22]. In addition to their intrinsic immune characteristics, γδ T cells also have adaptive immune functions [22]. According to the expression of the TCRδ chain, human γδ T cells can be divided into three cell subpopulations, Vδ1, Vδ2, and Vδ3T: Vδ1 T cells are mainly found in tissues; in contrast, Vδ2 T cells are mainly found in the peripheral blood and release a series of inflammatory factors [e.g., interferon gamma (IFN-γ) and tumour necrosis factor α (TNFα)] [23–25], and Vδ3 T cells constitute the smallest fraction of γδ T cells and are mainly distributed in the liver.

All subtypes of γδ T cells can exert cytotoxic effects, mediate tumor cell lysis, and secrete inflammatory factors to aid the activation of other immune cells for further antitumor effects [26–29]. Several single-cell RNA sequencing (scRNA-seq)-based studies have identified the presence of cytotoxic γδ T cells (γδ-CTLs) in tumor tissues [26, 30]. For example, Pizzolato et al. revealed the shared and unique cytotoxic characteristics of Vδ1 T and Vδ2γδ T cells by scRNA-seq [30]. Using scRNA-seq, Harmon et al. found that a subpopulation of Vδ1 T cells with cytotoxicity (high expression of GZMB, GZMK, IFN-γ, and TNF) is present in both endometrial carcinoma (EC) and colorectal cancer (CRC) [26]. Vδ1 T cells are the predominant γδ T-cell subset in human tissues and are found in mucosal tissues such as the dermis and intestinal epithelium. Vδ1 T cells induce apoptosis of tumor cells through cytotoxic mediators such as perforin and granzyme and by releasing IFN-γ and TNF-α [26]. The cytotoxic function of Vδ1 T cells has been used in a variety of cancer therapeutics [including acute lymphocyte leukemia (ALL), acute myeloid leukemia (AML), B-cell chronic lymphocytic leukemia (B-CLL), and neuroblastoma] [31–33]. Vδ2 T cells account for the largest proportion of tumor cells in the body and 2% to 5% of circulating CD3+ lymphocytes and are the predominant subpopulation of γδ T cells in the peripheral blood [33]. TCRs expressed by Vδ2 T cells preferentially couple with the Vδ2 and Vγ9 chains and directly target tumor cells via perforin and granzyme or indirectly target these cells through the release of IFN-γ and TNF-α [21, 34]. Vγ9Vδ2 T cells functionally share the characteristics of both αβT and NK cells, and these dynamic properties include receptor recombination, cellular memory, antigen presentation, and a non-MHC-restricted antibody-dependent cell-mediated cytotoxicity (ADCC) mechanism to mediate tumor killing [35].

### iNK-CTLs

Natural killer T cells (NKTs) are a specialized subpopulation of T lymphocytes that express both NKs (CD56 and CD161) and TCR-associated receptors, share some of their phenotypes and functions with NK cells and are components of the intrinsic immune system [2]. These cells are involved in the intrinsic immune response but also participate in and regulate the adaptive immune response. NKT cells can be classified into two types according to whether they respond to the α-galactosylceramide (α-GalCer)/CD1d complex: type I NKT cells (iNKT) and type II NKT cells respond and do not respond to this complex, respectively [2].

iNKT expresses a constant TCR composed of Vα24Jα18 chains (TCRα) and Vβ11 chains (TCRβ) [36]. CD1d, an MHC class I protein, is capable of presenting a variety of lipid antigens to T cells [37]. iNKT cells differentiate predominantly in the thymus into NKT1, NKT2, NKT17, and NKT10 cells [36, 38]. Studies have found that NKT1 cells tend to exhibit a higher level of cellular expression than other subpopulations of iNKT cells [38]. iNKT cells recognize aberrant cells, such as infected, damaged, senescent, and tumor cells that express a combination of lipid-CD1d molecules [37]. Upon stimulation by α-GalCer/CD1d, iNKT cells not only exhibit direct killing activity against tumor cells [39, 40] but also modulate other immune cells to exhibit indirect antitumor activity [41]. For example, CD1d on NSCLC induces iNKT cell-mediated cytotoxicity [37]. Konishi et al. found that α-GalCer/CD1d-stimulated NKT cells exert a direct killing effect on human lung cancer cell lines (RERF-LC-OK and PC-3) [42]. After activation, CD4-CD8-iNKT and CD4-CD8+ iNKT cells show cytotoxic function and increased IFN-γ production [38]. Another study found that iNKT cells are dependent on the perforin/granzyme pathway to mediate their killing effect on CRC [43].

## Cellular biomarkers of CTLs

Currently, there is a highly variable and incomplete agreement on the combination of biomarkers for identifying CTLs. In addition, some problems have been identified regarding biomarkers for CTLs, and these include the fact that lymphocytes that express molecules associated with cytotoxic functions are not necessarily cytotoxic [5]. Therefore, biomarkers for CTLs still need to be further explored and discussed in future studies. Here, we summarize the biomarkers for CTLs identified in previous studies and classify these into cell surface-related molecules, intracellular-related molecules and extracellular-related molecules [9, 44, 45].

### Cell surface-associated molecules

#### Lysosomal proteins LAMP-1 (CD107a) and LAMP-2 (CD107b)

The surface expression of the lysosomal proteins lysosome-associated membrane glycoprotein (LAMP)-1 (CD107a) and LAMP-2 (CD107b) may serve as one of the cellular markers of CTLs [5]. CTLs release cytotoxic particles against target cells that are secreted from secreted lysosomes that translocate and fuse to the plasma membrane [46]. After secretion of these particles, proteins located in the lysosomes, such as LAMP, LAMP-1 (CD107a), and LAMP-2 (CD107b), are abundantly expressed on the cell surface. These molecules are degranulation markers that can be used to recognize activated CTLs upon in vitro stimulation [5].

#### NK-associated surface molecules

NK-associated surface molecules, which were originally key receptors expressed in NK cells, have also recently been found to be potentially expressed on the surface of CD4+ CTLs and to be candidate markers of their cytotoxicity [14, 45, 47–52].

##### NKG7 [47, 48]

The potential of NKG7 as a marker of cytotoxicity in CD4+ CTLs has gained increasing attention in recent years. In the development of NKG7-Cre transgenic mice, NKG7 can be used to recognize CD4+ CTLs when crossing with Rosa26-LoxP-STOP-LoxP fluorescent reporter mice [47]. CD4+ CTLs expressing NK-related genes (e.g., Nkg7 and Klrb1) can be identified by scRNA-seq of peripheral blood mononuclear cells (PBMCs) [53]. Another scRNA-seq-based study found the presence of CD4+ CTLs coexpressing Gzmb and Nkg7 in bladder and liver cancers [54].

##### NKG2D [14, 49–52]

NKG2D serves as a key activating receptor expressed in NK cells, and it is thought that CD4+ T cells expressing NKG2D have a putative cytotoxic function independent of the TCR-MHC pathway [48]. Researchers initially identified NKG2D-expressing CD4+ CTLs in B-CLLs [44, 55]. CD4+NKG2D+ T cells are also thought to be cytotoxic cells and have been shown to be involved in rheumatoid arthritis (RA), Wegener’s granulomatosis (WG) and multiple sclerosis (MS), among other human autoimmune diseases [49–52, 56].

##### NKG2A, NKG2C/E, and SLAMF7 [14]

CD4+ CTLs can be recognized by NKG2A, a member of the C-type lectin receptor family, and form a heterodimer with CD94 [14]. NKG2C/E expression has been found on tissue-resident CD4+ CTLs from influenza A virus (IAV)-infected mice, and further studies have revealed that NKG2C/E expression in CD4+ CTLs is correlated with Blimp-1 expression and not with Eomesodermin (Eomes) expression [57]. SLAMF7 is significantly enriched in CD4+ CTLs, and in vitro studies have found that SLAMF7 expression increases MHC class II-dependent target cell killing [48].

#### Others

In recent years, numerous other molecules have been classified as biomarkers for CTLs, and these include CRTAM [14, 58], CD27, CD28 [14, 59], CD38 [60], CD26 [61] and CD56 [62–65]. CRTAM may receive increasing attention as a novel marker for CD4+ CTLs. CRTAM expression has been associated with enhanced cytolysis (e.g., Eomes, IFN-γ, GzmB, and perforin) [48]. Studies have found that partially activated CD4+ T cells express CRTAM, and only CRTAM+CD4+ T cells have the opportunity to develop into CD4+ CTLs [58]. CRTAM+CD4+ T cells can acquire cytotoxicity in response to interleukins (IL)-2 induction and are referred to as Th0 CTLs [14, 58]. In addition, CRTAM+ T cells can differentiate into Th1- or Th2-like cells and still retain their cytotoxicity [14]. The costimulatory receptors CD27 and CD28 are expressed at low levels on CD4+ CTLs and identify a highly differentiated T-cell phenotype [14, 59]. CD38, a glycoprotein with extracellular enzyme function, has a potentially cytotoxic function in malaria-infected individuals, as evidenced by the finding that CD38+CD4+ T-cell expansion is significantly correlated with a reduction in blood parasites [60]. CD26, a widely expressed glycoprotein with dipeptidyl peptidase IV (DPPIV) activity, has recently been proposed as a new marker for CD4+ CTLs [61]. CD56 expression may be correlated with the activation status of lymphocytes [62–64]. CD56+ γδ T cells exhibit enhanced antitumor cytotoxicity and have a strong IFN-γ production capacity [65, 66].

### Intracellular-related molecules

Based on scRNA-seq, researchers have found that KLRB1, KLRG1, KLRF1 and GPR56 may be able to serve as markers for CD4+ CTLs [67]. In addition, another study found that a subset of memory T cells with low expression of KLRG1 and high expression of CD127 (IL-7R) may serve as precursors of CD4+ CTLs [68]. High expression of the transcription factors RUNX3 and Eomes is also commonly used for the identification of CD4+ CTLs [48, 59].

### Extracellular-associated molecules

Cytotoxicity-associated molecules secreted by CTLs may also serve as cellular markers for CTLs, and these include granzyme A (GzmA), granzyme B (GzmB), granzyme K (GzmK) [45, 69], and perforin (Prf1) [3, 5, 45].

## Origin and differentiation trajectory of CTLs

**Fig. 1:**
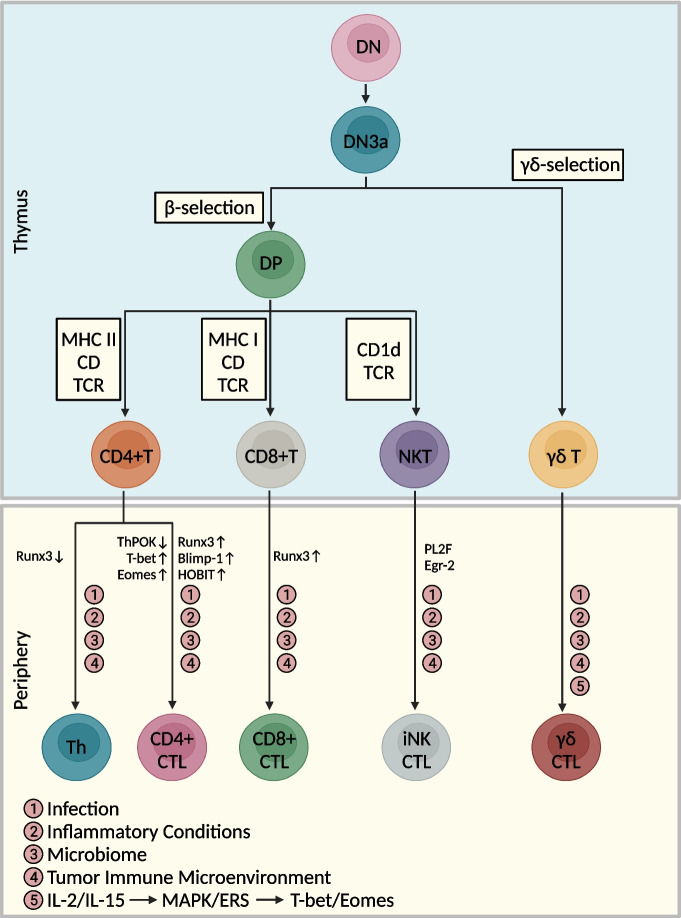
Differentiation trajectories of CTLs of thymic developmental origins and in the peripheral blood. This figure was created based on the tools provided by Biorender.com (accessed on 13/10/2023)

Thymic progenitor cells proliferate at the CD4(-) CD8(-) double negative (DN) stage. First, these cells enter the T-cell lineage at the DN2 stage, and this step is followed by completion of gene rearrangements at the TCRβ, TCRγ and TCRδ loci at the DN3 stage [70]. After β and γδ selection at the DN3a stage, these T cells enter the αβT and γδT lineages, respectively. Subsequently, the αβT lineage cells then downregulate CD25 and upregulate CD4 and CD8 to become double-positive (DP) cells. DP cells undergo TCRα gene rearrangement and the MHC-selection and CD1d-selection phases [70, 71], which gives rise to CD4+ T cells, CD8+ T cells and NKT cells. Most γδT lineage cells remain DN cells but downregulate CD24 expression upon maturation [70]. Studies have shown further activation of T-cell differentiation into CTLs under the influence of infection, inflammatory conditions, the microbiome, the tumor immune microenvironment (TIME), or stimulation of certain specific signaling pathways (Fig. 1).

### Differentiation pathways of CD4+ CTLs

**Fig. 2:**
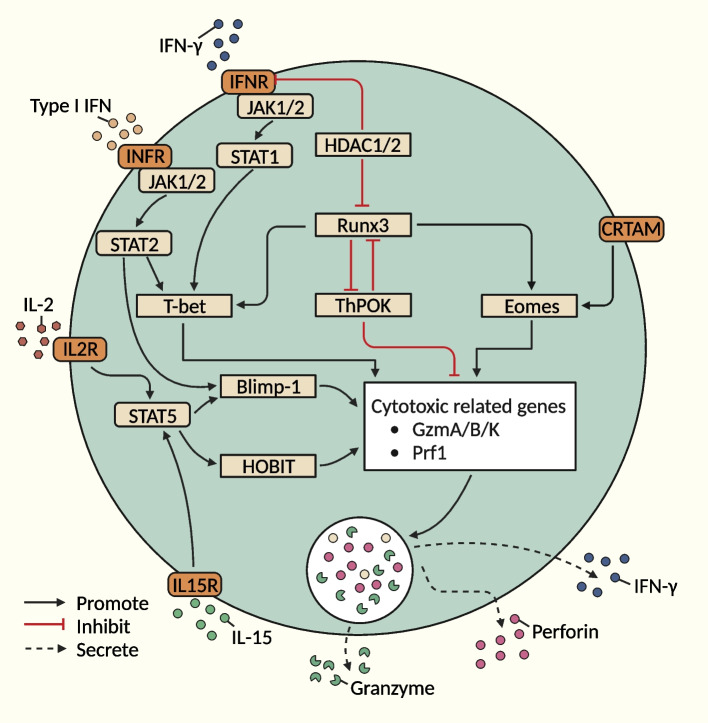
Differentiation trajectories of CD4+ CTLs. These mainly include I) dependence on TCR signaling as the initiating event pathway, (II) signaling through the receptor CRTAM as the initiating event pathway and (III) the epigenetic regulatory modification pathway. This figure was created based on the tools provided by Biorender.com (accessed on 13/10/2023)

CD4+ CTLs can further develop from the Th0, Th1, Th2, Th17 and Treg subpopulations [14]. At present, the transcription factors involved in the internal differentiation of CD4+ CTLs have not been fully unified and clarified. Transcription factors that induce cytotoxic effects in CD8+ CTLs [e.g., T-bet, B lymphocyte‐induced maturation protein‐1 (Blimp‐1), Eomes, RUNX Family Transcription Factor 3 (Runx3), T-helper inducing POZ-Kruppel like factor (ThPOK), and Homolog of Blimp-1 in T cells (HOBIT)] may be involved in the differentiation of CD4+ CTLs, but further validation is still needed [5, 44, 72, 73]. Studies have shown that CD4+ CTLs from Th1 cells represent the majority of CD4+ CTLs (predominantly secreting IFN-γ, TNF-α and IL-2) [14]. According to the current studies, the differentiation process of CD4+ CTLs may involve three pathways (Fig. 2): (I) dependence on TCR signaling as the initiating event pathway, (II) signaling through the receptor CRTAM as the initiating event pathway, and (III) epigenetic regulatory modification pathway [5, 55, 59, 74].

#### Dependence on TCR signaling as an initiating event

The thymus is a major site of T-cell development, and T-cell precursor cells originating from the bone marrow migrate to the thymus and differentiate into a variety of T-cell subpopulations [7]. TCRαβ thymocytes can differentiate into CD8+ CTLs, CD4+ Th cells, and NKT cells. The activity of key transcription factors controls the generation of a variety of Th profiles in response to a wide range of environmental signals. TCRαβ thymocytes can be differentiated into CD8+ CTLs, CD4+ Th cells, and NKT cells. Studies have shown that RUNX3/ThPOK, T-bet, Eomes, Blimp-1, and HOBIT play important roles in regulating the differentiation of CD4+ CTLs.

##### RUNX3/ThPOK transcription factor axis

Under appropriate stimulation (including TCR stimulation and IL-2/IL-15/type I IFN/IFN-γ), CD4+ Th cells can downregulate ThPOK for further differentiation into CD4+ CTLs [52, 55, 56, 73, 75]. The downregulation of THPOK is regulated by the RUNX3-THPOK silencing axis [59], which results in the upregulation of cytotoxicity-associated genes (e.g., Eomes, Ifng, GzmB and Prf1) [76]. Thus, the balance between ThPOK and RUNX3 expression may become a prerequisite for determining whether CD4+ Th cells differentiate into the CD4+ CTL lineage. In addition, IFN-γ ultimately mediates the expression of cytotoxicity-related molecules by phosphorylating signal transducer and activator of transcription (STAT)-1 (STAT1) and upregulating ThPOK [59].

##### Other transcription factors (T-bet, Eomes, Blimp-1, and HOBIT)

In addition to the RUNX3/ThPOK transcription factor axis, other transcription factors (e.g., T-bet, Eomes, Blimp-1, and HOBIT) can also be involved in regulating the differentiation of CD4+ CTLs [55]. For example, the binding of IL-2 to IL-2R further promotes the upregulation of Blimp-1/T-bet expression induced by STAT2 phosphorylation, which ultimately mediates the expression of cytotoxicity-associated molecules (e.g., IFN-γ, granzyme B and perforin) [5, 55, 59, 73, 75, 77, 78]. Furthermore, in the absence of STAT2, the expression of T-bet and granzyme B is reduced [59]. IL-2R on the surface of Tregs binds to IL-2 to competitively inhibit the differentiation of CD4+ CTLs [73]. Similar to T-bet, Eomes plays a key role in inducing the transcription of cytotoxicity-related genes in CD4+ CTLs [5, 55]. In addition, IL-15 binds to IL-15R on the surface of CD4+ T cells to activate HOBIT transcription induced by STAT5 phosphorylation, which ultimately mediates the expression of granzyme B and perforin in CD28-CD4+ T cells [79–81].

#### Signaling through CRTAM as an initiating event pathway

In addition to the RUNX3-dependent pathway, which relies on TCR signaling as an initiating event, CRTAM plays an important role in inducing the differentiation of CD4+ CTLs [14]. CRTAM directly regulates Eomes expression in an RUNX3-independent manner [14]. The previously identified roles of T-bet, Blimp-1, Eomes, Runx3, ThPOK, and HOBIT in regulating the expression of granzymes and other lysogenic molecules would be compatible with both the CRTAM-dependent and RUNX3-independent models of CD4+ CTL development [5].

#### Epigenetic modification pathways

In addition to transcription factor regulatory networks, epigenetic modification networks (e.g., DNA methylation and histone modification) play important roles in regulating gene expression. However, the current knowledge of epigenetic modifications in the regulation of CD4+ CTLs remains very limited. Protein acetylation modifications are controlled by histone acetyltransferase (HAT) and histone deacetylase (HDAC) and act as transcriptional coactivators and corepressors [59]. HDAC can be recruited to active gene loci, and in conjunction with HAT, this deacetylase further acts as a gene transcription regulator [59]. TCR activation with IFN-γ stimulation can induce JAK1/2 to further activate STAT1 [74]. HDAC1-deficient CD4+ T cells show increased levels of phosphorylated STAT1 (p-STAT1) [82]. Thus, HDAC1 can act as a key negative regulator of STAT1 activation in CD4+ T cells [82]. Later during T-cell development, HDAC1 and HDAC2 deficiency induces CD4+ Th cells to differentiate into CD4+ CTLs by upregulating RUNX3, and this differentiation is mainly manifested by upregulation of the expression of the CD8 gene profile (e.g., Cd8a and Cd8b1) [83]. In addition, HDAC1 and HDAC2 pass through and ultimately induce the generation of CD4+ CTLs [74]. Because STAT1 and STAT2 may be heterodimerized [84], the increased levels of phosphorylated STAT1 observed in HDAC1-HDAC2-deficient CD4+ T cells may indicate crosstalk between STAT1- and STAT2-dependent signaling pathways [59].

### Differentiation pathway of CD8+ CTLs

The process of generating CD8+ CTLs begins with hematopoietic stem cells (HSCs) in the bone marrow. HSCs mature and develop into common lymphoid progenitors (CLPs), and subsequently, CLPs migrate to the subperitoneal region of the thymus. Positive and negative selection processes in the thymus culminate in the development of CD4(-)CD8(+) T cells through the TCR binding and affinity for MHCI-like molecules [85]. Studies have shown that the IL-7 signaling pathway affects RUNX3 expression [86] and is critical for the generation of CD8+ CTLs [86–88]. In addition, the T-bet and Eomes transcription factors play important roles in the differentiation and function of CD8+ CTLs. T-bet and Eomes function together to induce the expression of IFN-γ, GzmB, perforin, CXCR3, and CXCR4 in CD8+ T cells, which ultimately mediate the generation of cytotoxicity in CD8+ T cells [55, 89]. Naïve CD8+ T cells are recruited to the draining lymph node (dLN) through CCR7 recirculation or chemokines such as CCR4/5. APCs process and present tumor antigens, migrate to the dLN and present antigens on its surface to naïve CD8+ T cells. The interaction between APCs and CD8+ T cells leads to the proliferation and activation of CD8+ T cells into CTLs, which downregulate CCR7 and upregulate chemokine receptors such as BLT1, CXCR3, CCR5, and CX3CR1. These cells then migrate to the TIME and ultimately mediate tumor cell killing [90].

### Differentiation pathway of γδ-CTLs

After γδ selection in the thymus, the γδ T lineage is differentiated. Most γδ T lineage cells remain DN cells but downregulate CD24 expression upon maturation [70]. Because γδ T cells remain immature in the thymus, γδ T cells function according to environmental signaling effectors in the periphery [27]. Upon antigen stimulation, γδ T lymphocytes shift from naïve (CD27+, CD62L+CCR7+, CD45RA+) cells to central memory cells with proliferative and low effector function (CD27+, CD62L+CCR7+, CD45RA-). Upon further antigen (Ag) stimulation, these cells further mature into effector memory cells (CD27-, CD45RA-) and produce IFN-γ or granzyme/perforin, which ultimately leads to lymphocytes expressing terminally differentiated expression of CD45RA (TEMRA) [30]. This maturation pathway from naïve to TEMRA cells was characterized in TCRVδ2+ γδ T cells, where TCR activation precedes and progressively drives the expression of cytotoxic receptors shared with NK cells [30]. Studies have shown that IL-2 and IL-15 induce the expression of CD107a (degranulation marker) on γδ T cells and give these cells their tumor cell-killing ability and that exogenous IL-2 and IL-15 also enhance the effector functions (especially degranulation/cytotoxic potential) of γδ T cells [91]. Further mechanistic investigations have revealed that IL-2/IL-15, through the mitogen-activated protein kinase (MAPK)/extracellular signal-regulated kinase 1/2 (ERK) pathway, induces the expression of T-bet and Eomes, which ultimately enhances the cytotoxic effects of γδ T cells [91].

### Differentiation pathways of iNK-CTLs

Similar to classical T-cell subsets, NKT cells develop in the thymus, but NKTs diverge as they enter the DP phase [71]. iNKT differentiation depends on the binding between the TCR and CD1d to initiate the NKT cell developmental program, which is further differentiated under early growth response 2 (Egr-2) and promyelocytic leukemia zinc finger (PLZF) selection [36]. iNKT cells can be divided into subpopulations similar to CD4+ Th cells. For example, NKT1 cells express T-bet and predominantly secrete IFN-γ, whereas NKT2 cells express GATA-binding protein 3 (GATA3) and PLZF and secrete Th2-type cytokines (e.g., IL-4 and IL-13). iNKT cells are also characterized by the expression of RAR-related orphan receptor-γ (RORγt) and the secretion of IL-17 [36]. Other subpopulations of NKT cells also exist, and these include IL-9-producing NKT cells, B-cell lymphoma 6 (BCL6)-expressing NKTFH, and IL-10-producing NKT10 cells [36]. T-bet is essential for the final maturation stage of iNKT cells and directly regulates the activation of genes associated with cytotoxicity in iNKT cells (e.g., perforin, CD178, and IFN-γ) [92, 93]. In contrast, T-bet-deficient iNKT cells are unable to produce IFN-γ in response to TCR stimulation and are unable to exhibit cytotoxic functions [92, 94].

## Cellular functions of CTLs in tumor immunity

**Fig. 3:**
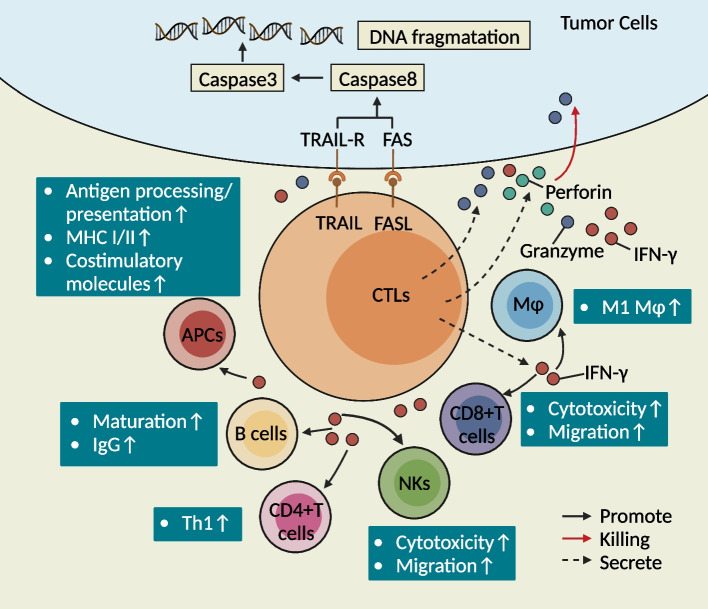
The main functions and pathways of the antitumor immune response mediated by CTLs in tumor cells mainly involve direct cytolytic killing and auxiliary activation of other immune cells to further mediate tumor killing. This figure was created based on the tools provided by Biorender.com (accessed on 13/10/2023)

Currently, the main focus on the function of CTLs is their killing effect on tumor cells (Fig. 3). On the one hand, CTLs mediate apoptosis in tumor cells mainly through the cytolytic action of granzymes/perforins or death receptor/ligand-dependent pathways. Recent studies found that the perforin/granzyme and death receptor/ligand systems could induce not only apoptosis in target cells but also other types of RCD, such as necroptosis and pyroptosis [3]. Furthermore, in addition to promoting apoptosis through granzyme-activated caspase activation in the perforin/granzyme system, the subsequent perforin-mediated Ca2+-dependent elevation of ROS and DNA damage processes may play an important role in promoting cell death [1]. On the other hand, CTLs may further exert their tumor-killing effects through interactions with other immune cells in the TME. In recent years, some studies have also suggested that CTLs may act as a “double-edged sword” in the tumor killing process and that these cells may also play a key role in promoting tumorigenesis and progression.

### Antitumor immunity

CD8+ CTLs, as the most classical and traditional CTLs, are mainly effector T cells that developed from activated naïve CD8+ T cells. After antigenic stimulation, CD27 and CD28 costimulatory receptors initiate signaling in CD8+ T cells. In addition, with the assistance of CD4+ T cells (CD40-CD40L interaction), CD8+ CTLs further exert their effects on tumor cell killing. Currently, the specific antitumor activity of CD8+ CTLs has been demonstrated to be effective against a variety of tumor types, such as melanoma, breast cancer, lung cancer, hepatocellular carcinoma (HCC), glioblastoma, acute and chronic leukemia, and lymphoma [7]. CD8+ CTLs directly exert cytolytic cell-killing effects and complementary activation of other immune cells to further exert tumor-killing effects. Mediating tumor cell killing plays a crucial role and has been associated with improved prognosis in tumor patients. For example, the significantly improved clinical prognosis of patients with proficient mismatch repair (pMMR) CRC is correlated with the extent of CD4+GzmB+ T-cell infiltration in the center of the tumor [95]. In addition, CD4+ CTLs are predictive of the outcome of patients with tumors treated with ICIs [54, 96–99]. CD4+ CTLs have also been shown to be important for controlling lung cancer metastasis [76]. CD4+ T cells kill melanoma cells in an MHCII-restricted manner after overt treatment with antigen-specific CD4+ T cells [16]. Th9/Th17 cells that were transferred to the host in a relayed manner induce tumor killing by releasing granzyme B [100, 101]. CD4+ T cells coexpressing chemokine (C-X-C motif) ligand (CXCL)-13 (CXCL13) and cytotoxic genes are associated with a significantly prolonged overall survival (OS) time in melanoma patients [102]. Naïve CD4+ T cells can further differentiate into CD4+ CTLs and mediate the killing of melanoma cells in lymphocytopenic host bodies [16]. Currently, studies on the function of γδ-CTLs have focused on antitumor immunity, mainly through their direct cytolytic killing and adjunctive activation of other immune cells for further tumor killing [27]. For example, Vδ1+ T cells are highly cytotoxic against neuroblastoma [103]. Vδ1+ T cells isolated from tumor infiltrating lymphocytes (TILs) in colon tumors are cytotoxic to both autologous and allogeneic epithelial tumor cells [103]. In addition, the infiltration of Vδ2 CTLs (CD107a+) is associated with favorable clinical outcomes in bladder cancer patients and induces enhanced secretion of IFN-γ and TNF-α. iNK-CTLs, as important players in cytotoxic functions, play an indispensable role in mediating tumor cell killing and improving tumor prognosis. Studies have demonstrated a significant correlation between an increased number of IFN-γ-producing iNK-CTLs and prolonged survival in NSCLC patients [41]. High infiltration of iNK-CTLs in the TME significantly improves the 5-year recurrence-free survival (5y-RFS) of stage III CRC patients [104].

#### Direct lysogenic-type killing effects

**Fig. 4:**
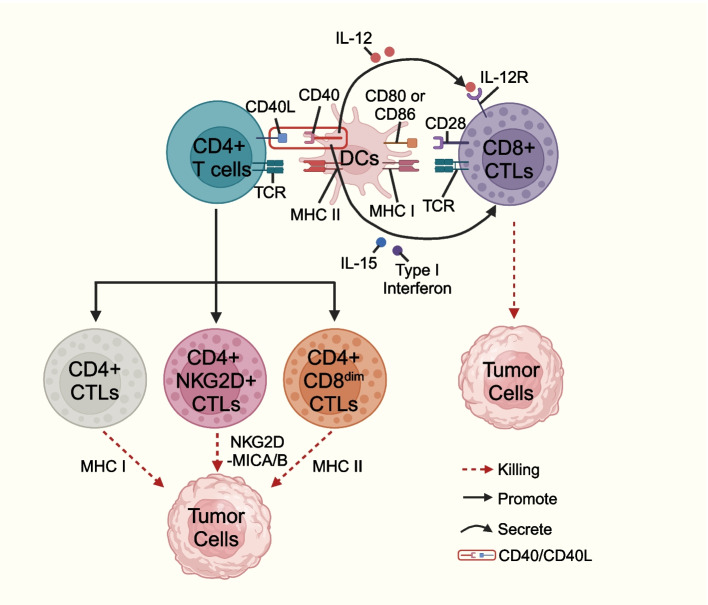
Key cell surface receptor-ligand interactions between the CD4+CTLs, CD4+T cells, DCs, CD8+ CTLs, and tumor cells. This figure was created based on the tools provided by Biorender.com (accessed on 21/02/2024)

The granzyme/perforin pathway and death receptor-dependent pathway are of great importance in exerting direct cytolytic-type killing effects [105–107]. After specific TCR signaling, CTLs secrete perforin to lyse target cell membranes, and granzymes undergo cytosolization with the help of perforin and translocate to target cells to induce apoptosis [108]. The death receptor-dependent pathways in CTLs mainly include Fas/FasL and TRAIL/TRAIL-R [105, 106]. FASL expressed on the surface of effector cells binds to FAS on the surface of target cells and activates the intracellular Fas-associated death domain (FADD)/caspase8/FADD-like IL-1b-converting enzyme-like apoptotic protein-inhibitory protein (FLIP)-induced death signaling complex and eventually caspase3-mediated apoptosis in target cells [18]. TRAIL expressed on the surface of CTLs binds to TRAIL-R on the surface of target cells and can exert a killing effect on tumor cells that are resistant to the Fas/FasL pathway [106]. Recent studies found that the granzyme/perforin pathway and death receptor-dependent pathway selection are affected by exogenous stimulus signal intensity and the local microenvironment; for example, under conditions consisting of a high concentration of a specific antigen and the absence of IL-2, CD4+ CTLs prefer to adopt the Fas/FasL pathway for the killing of target cells, and under conditions consisting of a low antigen concentration in the presence of IL-2, CD4+ CTLs prefer to utilize perforin/granzyme pathway-mediated killing [109]. CD4+ CTLs may kill tumor cells through three potential mechanisms (Fig. 4): First, CD4+ CTLs can recognize homologous antigens presented by APCs and secrete granules to kill target cells in the MHC class II-dependent manner; Second, CD4+ CTLs can upregulate NKG2D to kill tumor cells in the NKG2D-MICA/B pathway-dependent manner; Third, CD4+CD8dim CTLs expressing low levels of CD8 (CD8dim) can kill tumor cells in a MHC class I-dependent manner [55]. CD8+ T cells can initiate subsequent cytotoxic effects upon antigenic stimulation. Additionally, CD4+ Th can activate gene expression programs in CD8+ CTLs and enhance their function. DCs present antigens to CD4+ Th in the context of MHC class II, leading to increased expression of CD40L on the surface of CD4+ Th. Subsequent binding of CD40L on CD4+ Th and CD40 on cDC1 further enhances antigen presentation capacity of cDC1 (e.g. MHC class I molecules) as well as expression of costimulatory ligands (e.g. CD80 and/or CD86 and CD70) and cytokines (e.g. type I interferons, IL-12, and IL-15). cDC1s then mediate the cytotoxicity of CD8+ CTLs against target cells in a MHC class I-dependent manner (Fig. 4) [110]. Recently, CD5+ DCs have been found to induce anti-CD4+ Th and anti-CD8+ CTL responses against tumors and enhance responses to immunotherapy [111]. Vδ1+ and Vδ2+ γδ T cells recruited to the TME further exert tumor killing effects through perforin/granzyme-, IFN-γ/TNF-α-, death receptor ligand-, and ADCC pathway-mediated cytotoxicity [27, 34]. γδ T cells can be targeted to tumors via ADCC, where CD16 (Fcγ receptor III) expressed on γδ T cells binds to the target cells with the antibody’s Fc fragment to mediate tumor killing [112, 113]. iNK-CTLs can exert cytotoxicity through the Fas/FasL pathway, the TNF-α pathway, and the granzyme and perforin pathways of tumor cells carrying CD1d molecules [37, 114]. The Fas/FasL antitumor pathway in iNK-CTLs cells causes apoptosis through the effector cell’s action of FasL on the surface of iNK-CTLs cells with cells expressing Fas molecules, which destroys tumor cells. The killing effect of TNF-α requires prolonged contact between effector cells and target cells to further promote perforin protein activity, resulting in effective killing of most tumor cells. Studies have shown that for tumor cell elimination, the contribution of perforin/granzyme is more significant than that of Fas/FasL [115]. Similarly, α-GalCer-activated iNK-CTLs exert cytotoxic activity via perforin/granzyme [37].

#### Adjuvant activation of other immune cells

CTLs further exert an adjuvant antitumor immune response by secreting inflammatory factors (e.g., IFN-γ) and enhancing the function of immune cells such as CD8+ CTLs, B cells, macrophages, DCs, NKs, and APCs [116].

##### Macrophages and APCs

Macrophages are more inclined to transform into M1-type macrophages stimulated by IFN-γ secreted by CTLs and participate in Th1 antigen-specific responses [117, 118]. M1-type macrophages are proinflammatory and exert antigen-presenting functions, which further induces cytotoxicity in CD8+ CTLs. IFN-γ produced by CTLs can upregulate the expression of TAP-1 and TAP-2, which can further upregulate the antigen processing and presentation functions of APCs. In contrast, IFN-γ can also increase the expression of MHC class I and MHC class II proteins [116]. In addition, transduction of the IFN-γ signaling pathway contributes to upregulation of the expression of costimulatory molecules on the cell surface of APCs, which enhances their involvement in cellular immunity [116]. For example, IFN-γ secreted by iNK-CTLs promotes the upregulation of costimulatory molecules and MHC class II molecules by DCs, and subsequently, IFN-γ induces IL-12 production in a CD40/CD40L-dependent manner [116–118]. Sustained IL-12 secretion by mature DCs triggers iNK-CTLs cells to increase IL-12R expression and thereby promotes a positive feedback loop between iNK-CTLs and DCs [2]. Activated Vγ9Vδ2+ T cells can promote the maturation of DCs by secreting cytokines (e.g., IFN-γ and TNF-α) [34]. In addition, IFN-γ and TNF-α secreted by Vγ9Vδ2+ T cells could promote DCs to upregulate CCR7 and facilitate DCs to migrate to lymphoid tissues to activate CD4+αβ T cells and thus initiate the immune response [34, 119]. IFN-γ could help DCs further upregulate the expression of MHC molecules and costimulatory molecules, which could further enhance their functions in antigen processing and presentation [116]. All of the abovementioned mechanisms facilitate the immune elimination of tumor cells [116].

##### B cells

IFN-γ produced by CTLs is important for promoting B-cell proliferation and regulating antibody class switching [12, 34, 116, 117, 120, 121]. IFN-γ binds to B-cell receptors and CD40 activation signals to induce BCL-6 expression. IFN-γ and IL-12 synergistically promote antibody class switching from IgM to IgG2a (a higher-affinity specific antibody) and thereby facilitate the processing and presentation of Ag by other immune cells [117]. For example, CXCR5+CD8+ T cells [122] with cytotoxic functions exhibit B-cell helper functions, which mainly include stimulation of B-cell proliferation, antibody/B-cell receptor (BCR) class switching and antibody production [12, 120, 121], and enhancement of CD4+ Th-B-cell interactions [12]. In addition, another study found that CXCR5+ICOS+CD8+ T cells show significant infiltration in tumor lymph nodes (LNs) of patients with Hodgkin’s lymphoma (HL) and could upregulate the expression of IL-2, IL-4, and IL-21 [123], which promotes B-cell proliferation and antibody production. In addition, IFN-γ secreted by Vγ9Vδ2 T cells plays an important role in regulating B-cell maturation and immune-antibody production [34, 117]. iNK-CTLs can form a bidirectional interaction with B cells: on the one hand, B cells can present lipid antigens to type I NKT cells via CD1d [124], and on the other hand, iNK-CTLs can license B cells to effectively initiate and activate the antitumor response [2].

##### CD8+ CTLs, NKs and CD4+ Th cells

The stimulation of CD8+ CTLs by IFN-γ signaling upregulates the expression of IL-2R, T-bet and granzyme, which serve as important mediators for tumor killing by CD8+ CTLs [116]. In addition, IFN-γ mediates the migration of CD8+ CTLs and NKs to the TME by promoting the expression of chemokines (e.g., CXCL9, CXCL10, and CXCR3) [125], which ultimately enhances the cytotoxic effect of CD8+ CTLs and inhibits tumorigenesis and progression [117]. IFN-γ acts on NKs and promotes the killing of tumor cells by NKs through TRAIL, whereas TRAIL expression can be enhanced by IFN-γ-induced IRF1 [37, 93, 117]. In addition, in response to antigen re-exposure and activation of cytokine release, the antigen-specific memory of CTLs releases a variety of cytokines and chemokines, such as IFN-γ, CC motif chemokine ligand 3 (CCL3), and monocyte chemoattractant protein-1 (MCP1), which contribute to the recruitment of monocytes and NK cells and upregulate the secretion of CXCL9 and CXCL10 to further recruit NKs to further activate B cells and DCs [34, 117]. In addition, IFN-γ secreted by CTLs plays an important role in mediating the differentiation of Th1 cells, which enhances the antitumor effects in the TME [99, 117, 118].

#### Promotion of tumorigenesis and progression

Immunosuppressive CD4 T-cell subsets (e.g., Tr1) have been shown to have cytotoxic functions [126, 127] but may play a role in promoting tumorigenesis and progression. For example, cytotoxic Tr1 cells can counteract tumor immune responses through their killing effect on APCs [128]. Cytotoxic GZMK+Eomes+Tr1 is associated with tumor progression in CRC, non-small cell lung cancer (NSCLC), and tumors that develop liver metastases [96, 129]. In addition, IL10 secreted by Tr1 cells may promote the transformation of macrophages to M2-type macrophages and inhibit the maturation of DCs to further play a role in promoting tumor growth [96, 130, 131].

## Improving the cytotoxic function of CTLs

Cytotoxic CTLs induce a range of different types of damage in tumors, including necrosis, apoptosis, necrotic apoptosis, and cellular pyroptosis [132]. However, tumor cells initiate a series of pathways to downregulate the cytotoxicity of CTLs, which will help tumor cells evade recognition and killing by the immune system [132]. Therefore, understanding how tumor cells regulate the cytotoxicity of CTLs provides a theoretical basis for improving the cytotoxicity of CTLs and for enhancing the role of CTLs in antitumor immunity. Studies have shown that the cytotoxicity of CTLs could be improved or enhanced by modulating the expression level of cytokines, reducing the infiltration ratio of certain specific immune cells, modulating the expression level of certain molecules in the TIME, or altering certain metabolic pathways in CTLs.

### CD4+ CTLs

Xu et al. found that anti-PD-1-IL-15m improves tumor-infiltrating T-cell function and antitumor immunity, and anti-PD-1-IL-15m enhances the proliferative capacity and cytotoxicity of CD8+ TILs and CD4+ TILs, but the underlying molecular mechanism has not been clarified [133]. IFN-γ upregulates the expression of MHC II and increases the cytotoxic effect of CD4+ CTLs on tumor cells [134]. The blockade of HLA-G/CD85j increases the cytolytic activity of CD4+ CTLs to improve the antitumor immune responses [135, 136]. Tregs utilize IL-2 deprivation to inhibit T-cell-mediated cellular immunity, whereas endogenous IL-2 drives the upregulation of the transcription factor Blimp-1 within CD4+ Th cells to further promote granzyme B expression, and Tregs may compete with IL-2 to negatively control this process [73]. CD137 stimulation induces increased expression of cytotoxicity program markers (Eomes/Granzyme B) in Tregs while maintaining Foxp3 properties, and CD137 agonist therapy reprograms Tregs to CD4+ CTLs [137].

### CD8+ CTLs

In mouse models, the combination of OX40 costimulation and the PD-1 inhibitory pathway promotes the coexpression of multiple NK cell receptors (e.g., NKG2A, NKG2D, and KLRG1) and chemokine receptors by CD4+ T cells and CD8+ T cells, which have high potential to proliferate and exhibit cytotoxicity [138]. OX40-activated CD4+ T cells may also contribute to CD8+ T-cell expansion and differentiation [138]. In a mouse model, CCL21+ICAM1 enhances the cytotoxicity of CD8+ T cells, as evidenced mainly by a significant increase in the granzyme B levels [139]. In addition, oncolytic herpes simplex virus 1 (oHSV) carrying CD40L enhances the maturation of DCs in the TME, promotes Th1 differentiation, and enhances the cytotoxicity of CD8+ CTLs [140]. In B-CLL, activated CD4+ Th cells increase miR-181b expression in B-CLL via CD40-CD40L signaling, mediate a decrease in IL-10 expression, and further enhance the cytotoxicity of CD8+ CTLs [141]. In a mouse melanoma model, treatment using a combination of CD47 and CTLA4 blockade with radiotherapy (RT) results in a significant increase in CD8+ CTLs in mouse tumors [142]. Blockade of the tumor-associated macrophage (TAM) scavenger receptors MARCO and IL37R reduces the number of Tregs and restores the cytotoxicity and antitumor capacity of NKs and CD8+ CTLs [143]. Clec9A on cDC1 increases the cytotoxic effect of CD8+ CTLs [144]. The enhancement of acetate metabolism in CD8+ CTLs could enhance the efficacy of CD8+ CTLs [145]. Studies have shown that LSD1 forms nuclear complexes with Eomes of CD8+ CTLs from immunotherapy-resistant melanoma and breast cancer patients, ultimately mediating dysfunction of CD8+ CTLs [146, 147], and targeting the phosphorylation of the LSD1 pathway can increase the cytotoxicity of CD8+ CTLs [147]. Activated CD8+ CTLs exhibit upregulation of the glycolytic pathway and require CD28 costimulatory signaling to prolong the duration of glycolytic upregulation; in an obese mouse model of breast cancer, the knockdown of STAT3 in CD8+ CTLs or treatment with inhibitors of fatty acid oxidation increases both glycolysis and the toxic function of CD8+ CTLs (including IFN-γ, granzyme B and CD107a) and thereby inhibits mammary tumor development [148].

### γδ-CTLs and iNKT-CTLs

Vδ1 T cells are adapted to the TIME of hypoxia, and in vitro studies have shown that culturing Vδ1 T cells under hypoxic conditions enhances their cytotoxicity [149]. IL-2/IL-21 significantly promotes the proliferation and cytotoxic function of γδ T cells [150, 151]. Blocking TIM-3 increases the killing effect of Vγ9+Vδ2+ T cells on colon cancer cells by activating the ERK1/2 pathway and upregulating perforin and granzyme B expression [152]. In addition, the activation of BTN3A/CD277 promotes the activation and enhances the cytotoxicity of γδ T cells [153]. iNKT-CTLs are reduced in number and functionally impaired in many cancer types, and the underlying causes may involve loss/downregulation of CD1d expression, loss of β2-microglobulin, or lack of activating antigens [104]. However, studies on increasing the cytotoxicity of iNKT-CTLs remain very limited.

## Open questions

### The definition of CTLs needs to be rethought

Different studies have established different criteria for defining CTLs. In the past, CTLs were considered a group of effector cells that differentiated from initial CD8+ T cells after activation and exerted direct killing effects on target cells. In recent years, with the development of high-throughput sequencing, more molecular signatures of cytotoxicity have been identified based on cell surface biomarkers. Currently, the definition of CTLs is not only limited to CD8+ T cells with cytotoxicity but also includes CD4+ T cells, NKT cells and γδ T cells that can exhibit cytotoxic functions. In addition, no uniform and standardized biomarkers have been established for determining CTLs. On the one hand, some T cells expressing molecules related to cytotoxic function do not necessarily exhibit cytotoxic function. For example, CD8+ T cells expressing GzmK and GzmB only exhibit very low cytotoxicity potential and do not exert sufficient cytotoxicity to kill target cells. On the other hand, cytotoxicity markers such as cytotoxic degranulation molecules, granzyme- and perforin-encoding genes, cytotoxicity differentiation-associated transcription factors, markers associated with cellular signaling, NK cell surface receptor molecules, CRTAM, and transcription factors (Eomes and RUNX3) have not yet been consistently identified by different studies. Therefore, many future studies are needed to demonstrate whether specific markers can become the gold standard for determining CTLs.

### The functional subtypes of CTLs in different T-cell subtypes need to be further explored

With the widespread use of single-cell sequencing, an increasing number of CTL subtypes have been identified. For example, several scRNA-seq-based studies have identified the presence of cytotoxic γδ-CTLs in tumor tissues [26, 30], and through scRNA-seq, researchers have identified the presence of cytotoxic Vδ1 T-cell subpopulations in both EC and CRC [26]. Another scRNA-seq-based study found CD4+ CTLs coexpressing Gzmb and Nkg7 in bladder and liver cancers [54]. CD4+ CTLs expressing NK-associated genes were identified in PBMCs by scRNA-seq [53]. However, whether cytotoxic cells are also present in other T cells has not been clarified. In addition, the following issues remain unaddressed in the field of CTLs: 1) whether different CTLs can be categorized into different subtypes and 2) the biomarkers, functional identification and validation of different subtypes of CTLs need to be further improved by many studies.

### The key molecules involved in the differentiation of different CTLs are controversial

Currently, the key targets regarding the differentiation trajectories and differentiation nodes of CTLs have not been fully unified. On the one hand, the differentiation trajectories regarding CD4+ CTLs have not been fully clarified and are currently divided into (I) TCR signaling as the initiating event pathway, (II) signaling through the receptor CRTAM as the initiating event pathway, and (III) epigenetic regulatory modification pathway. However, the existence of other pathways that could mediate the differentiation of CD4+ CTLs remains unclear. In addition, our understanding of the role of epigenetic regulatory modifications mediating the differentiation of CD4+ CTLs remains very limited. On the other hand, the differentiation pathways of NK-CTL and Vδ-CTL in CD8+ CTLs remain the more traditional differentiation pathways, including transcription factor regulation (e.g., T-bet, Eomes, Egr-2, and PLZF) and peripheral antigenic stimulation. Identifying the key molecules that regulate CTL differentiation is beneficial for the regulation and intervention of CTL differentiation, and whether targeted drugs exist for these key molecules remains unclear. Further preclinical studies are needed to further explore this issue in the future.

### The multiomics characterization of different subtypes of CTLs remains unclear

Different subtypes of CTLs may have different multiomics features (e.g., proteomics, metabolomics, transcriptomics, genomics and epigenomics). Based on metabolomics, activation of the acetate metabolic pathway could further mediate the stronger cytotoxic function of CD8+ CTLs [145]. Activation of the glycolytic pathway facilitates the activation of CD8+ CTLs for further subsequent cell killing functions [148]. The hypoxic environment could promote γδ T cells to further enhance their cytotoxic function [149]. Based on transcriptomics, the expression of some cytokines (e.g., IL-2/IL-21) can significantly promote the proliferation and cytotoxic function of γδ T cells [150, 151]. Endogenous IL-2 drives the upregulation of the transcription factor Blimp-1 within CD4+ Th cells, which further promotes the expression of GzmB and thereby drives the differentiation of CD4+ Th cells to CD4+ CTLs [73]. CD137 induces an increase in the expression of cytotoxic molecules in Tregs while preserving Foxp3 properties [137]. However, much research is still needed to further discover whether other subtypes of CTLs may have different multiomics profiles.

The regulation and management of cytotoxic functions of CTLs based on multiomics profiling maximizes the tumor-killing effects of CTLs. CD137 agonist therapy promotes the conversion of Tregs into CD4+ CTLs [137]. Upregulation of the acetate metabolic pathway could enhance the efficacy of CD8+ CTLs [145]. Targeting the phosphorylated LSD1 pathway increases the cytotoxicity of CD8+ CTLs [147], and inhibition of fatty acid oxidation can increase glycolysis to further enhance the toxic function of CD8+ CTLs [148]. Therefore, strategies for managing and regulating the cytotoxic function of CTLs based on these multiomics features remains an important direction for future research.

### The relationship between microorganisms (intratumoral or intestinal) and CTLs is unclear

Studies have identified microorganisms that may have an important influence on the function of CTLs. In terms of intratumoral microorganisms, Talimogene laherparepvec (T-VEC) [a genetically modified type-I herpes simplex virus] can mediate the recruitment of CD8+ CTLs to the TME by modulating the secretion of type-I IFNs and chemokines (e.g., CXCL9 and CXCL10) and thereby triggering *cytotoxic* tumor-killing effects [154]. In addition, some of the intratumoral viral microbes are associated with increased NKT cell infiltration and significantly improved prognosis of tumor patients [155]. In melanoma, Lactobacillus spp. are positively correlated with the abundance of CD8+ CTLs, and the infiltration of CD8+ CTLs could progressively increase by increasing the abundance of *Chlamydia trachomatis* within the tumor [154]. Intratumoral Clostridium spp. and their associated metabolites can further exert tumor-killing effects by upregulating caspase3 and activating CD8+ CTLs [156]. Several studies have attempted to elucidate the effects of intratumoral microorganisms on CTLs, but these limited studies cannot fully elucidate the effects of intratumoral microorganisms on CTLs. Therefore, many studies are still needed to further explore the role of intratumoral microorganisms on CTLs in the future.

However, the understanding of the impact of intestinal microbes on the production of CTLs is much more limited. Intestinal microbes may influence the proportion of CD8+ CTLs that infiltrated cutaneous melanomas, and Lachnoclostridium is positively correlated with the expression of infiltrated CD8+ CTLs and the chemokines CXCL9, CXCL10, and CCL5 in cutaneous melanoma tissues [154]. In addition, studies have shown that intratumor microbes and intestinal microbes play interrelated and interacting roles [157], and therefore, future attention needs to be paid to the effects of intestinal microbes on CTLs.

At present, the number of studies on the effect of microorganisms on the antitumor mechanism of CTLs remains very limited. Based on the abovementioned studies, we found that the tumor killing mechanism of microorganisms on CTLs mainly involves the regulation of cytokine secretion and the regulation of cellular chemotaxis. However, these mechanisms are not sufficient to fully elucidate the effect of microbes on the antitumor mechanism of CTLs. Therefore, future research in this direction is still needs to further strengthen and consolidate the conclusions. In addition to the effects of microorganisms on CTLs, other immune cells and stromal cells still exist in the TIME. The crosstalk between immune and stromal cells, which are important components of the TIME, and microbes has not been systematically explored. Therefore, this issue still needs to be addressed in and be the focus of future studies.

### The aging patterns of different types of CTLs and strategies for reversing the aging process of CTLs have not yet been elucidated

Immunosenescence decreases the body’s immune surveillance and immune clearance abilities, resulting in a restricted immune response and tumorigenesis. Cellular senescence refers to a permanent cell cycle arrest state in which cells lose their ability to divide, and this effect is often accompanied by upregulation of cytokine expression and enhanced cellular secretion [158]. Cellular senescence can occur at all stages of growth and development and is an important mechanism for maintaining tissue homeostasis and preventing the expansion of damaged cells [159]. Cellular senescence can alter the adaptability of immune cells and ultimately affect the outcome of cancer therapy [160]. Senescent T cells, as late differentiated memory/effector T cells, lack CD28 expression but express CD57 and regulatory receptors [161, 162]. In addition, senescent T cells express CD45RA but not CD45RO and are in cell cycle arrest [161, 162]. Studies conducted in recent years found that a senescence pattern also exists in CTLs found in the TME [162, 163]. Shosaku et al. revealed that CTLs presenting epigenetically enhanced enhancers and repressed promoters imply a senescence pattern of CTLs [163]. However, few studies have attempted to explore the mechanisms mediating the senescence states and senescence patterns of CTLs. Therefore, the senescence pattern based on CTLs remains an important direction for future antitumor immunity. In addition, there remain more unanswered questions regarding the senescence of CTLs. First, the distinction between different subtypes regarding the senescence of CTLs remains unclear. Second, drugs that intervene with the senescence targets of CTLs can be discovered in the future to precisely regulate and manage the senescence patterns of CTLs or their specific subtypes.

### The epigenetic regulation of CTLs is unclear

Our current understanding of the epigenetic regulation of CTLs heterogeneity, plasticity, and dysfunction remains rudimentary. While key transcription factors driving CTLs differentiation are being mapped out, how epigenetic modifications shape diverse CTLs subpopulations and control their fate in the complex tumor microenvironment is poorly defined. Future studies should conduct integrated multi-omics profiling of DNA methylation, histone modifications, chromatin accessibility, and gene expression in intratumoral CTLs compared to healthy CTLs to reveal dysregulated epigenetic patterns associated with exhaustion. Genetic and pharmacological perturbation of epigenetic regulators in mouse models can help causally evaluate the impact on CTLs accumulation, subtype composition, and *cytotoxic* functions within tumors. By mapping epigenetic landscapes linked to CTLs heterogeneity and impairment, we can identify novel drug targets to reverse maladaptive epigenetic programming and reinvigorate anti-tumor immunity. Single-cell multi-omics approaches combining ATAC-seq, ChIP-seq and RNA-seq will provide further resolution of the epigenetic circuitry orchestrating CTLs divergence and dysfunction. Finally, elucidating interactions between epigenetic alterations, transcriptional networks, and metabolic pathways offers systems-level insight into how extrinsic signals shape CTLs identity and adaptive fitness in the tumor microenvironment. Comprehensively elucidating the epigenetic underpinnings of CTLs properties and fate decisions will uncover new strategies to combat CTLs dysfunction and improve immunotherapies.

## Data Availability

No datasets were generated or analysed during the current study.

## Abbreviatons

CTLs: Cytotoxic T Lymphocytes
CD: Cluster of Differentiation
NK: Natural Killer
γδ T cells: Gamma Delta T cells
TCR: T-cell Receptor
APCs: Antigen-Presenting Cells
HSCs: Hematopoietic Stem Cells
CLPs: Common Lymphoid Progenitors
iNKT: Invariant Natural Killer T
scRNA-seq: Single-cell RNA Sequencing
TME: Tumor Microenvironment
IFN-γ: Interferon-gamma
STAT: Signal Transducer and Activator of Transcription
ROS: Reactive Oxygen Species
RCD: Regulated Cell Death
AML: Acute Myeloid Leukemia
ALL: Acute Lymphocytic Leukemia
CRC: Colorectal Cancer
NSCLC: Non-Small Cell Lung Cancer
B-CLL: B-Cell Chronic Lymphocytic Leukemia
ADCC: Antibody-Dependent Cell-Mediated Cytotoxicity
RORγt: RAR-related Orphan Receptor-gamma
BCL6: B-Cell Lymphoma 6
HDAC: Histone Deacetylase
LSD1: Lysine-Specific Demethylase 1
T-VEC: Talimogene Laherparepvec
IL: Interleukin
CXCR: Chemokine (C-X-C motif) Receptor
DCs: Dendritic Cells
TAM: Tumor-Associated Macrophage
TIME: Tumor Immune Microenvironment
TNFα: tumour necrosis factor α
MHC: major histocompatibility complex
Th: T helper cells
RUNX3: RUNX Family Transcription Factor 3
Eomes: Eomesodermin
LAMP: lysosome-associated membrane glycoprotein
Blimp-1: B lymphocyte-induced maturation protein-1
Runx3: RUNX Family Transcription Factor 3
ThPOK: T-helper inducing POZ-Kruppel like factor
HOBIT: Homolog of Blimp-1 in T cells
CXCL: Chemokine (C-X-C motif) ligand
